# Development and Validation of a LC-MS/MS Method for the Determination of Nitrofuran Metabolites in Soft-Shell Turtle Powder Health Food Supplement

**DOI:** 10.1155/2021/8822448

**Published:** 2021-03-08

**Authors:** EunChae Ryu, Ji Sung Park, Sib Sankar Giri, Se Chang Park

**Affiliations:** ^1^Laboratory of Aquatic Biomedicine, College of Veterinary Medicine and Research Institute for Veterinary Science, Seoul National University, Seoul 08826, Republic of Korea; ^2^Seoul Regional Office, Animal and Plant Quarantine Agency, Seoul 07670, Republic of Korea

## Abstract

Soft-shell turtle (SST; freshwater terrapin or tortoise) is a popular and important health functional food (HFF) product in many Asian countries. HFFs containing SST must be safe, but several HFFs have been found to be contaminated with dangerous substances, such as nitrofuran metabolites (NFMs). This finding suggests that the consumption of HFFs results in the regular exposure of vulnerable individuals to hazardous substances. Importantly, nitrofuran antibiotics have been banned for use in food-producing animals since the 1990s by the European Union. Thus, in this study, we propose a reliable and quick method to reduce the time required for the detection of four NFMs in SST powder that conventional methods are unable to quantify. Our method involves the derivatization and hydrolysis of SST powder and was validated in accordance with the requirements of European Commission Decision 2002/657/EC. The method achieves an apparent mean recovery of 82.2–108.1%, repeatability of 1.5–3.8%, and reproducibility of 2.2–4.8% for 0.5–10.0 *μ*g kg^−1^ of 1-aminohydantoin, semicarbazide, 3-amino-2-oxazolidinone, and 3-amino-5-morpholinomethyl-2-oxazolidinone. In addition, linearity was achieved with correlation coefficients of 0.999, and the detection capability (CC*β*) and decision limit (CC*α*) were found to be reliable, indicating that this is a fast and accurate method for the analysis of SST powder. The validated method was successfully applied to detect NFMs in SST powder in commercial HHFs.

## 1. Introduction

Currently, health functional foods (HFFs, also known as health-promoting foods or health beneficial foods) represent the fastest growing area in the food industry and health food market [1]. In particular, the production of HFFs containing plant or animal extracts is rapidly gaining importance [2]. In this context, soft-shell turtle (SST; freshwater terrapin or tortoise) products are sold as dietary supplements, as well as for traditional Chinese medicinal purposes. Typically, SST is sold in powdered form in capsules or liquid form and is available from both traditional and online markets. SST is, thus, an important commercial product and is a popular delicacy in East and Southeast Asian countries for its nutritional and medicinal properties [3, 4]. The production of Chinese SST reached 331,424 tons in 2012, and sale as a dietary product was the main commercial use [5]. SST has a high nutritional content and is known for the beneficial effects in preventing osteoporosis and bone loss in old age, promoting childhood development, and improving conditions such as back pain, diabetes, allergy, hypertension, asthma, and thyroid disorders, as reported by practitioners of traditional Chinese medicine, which has resulted in its increased popularity and scale of farming [6–8].

The use of antimicrobial veterinary medicines is necessary to rear animals profitably in an intensive fashion, but the use of these products can result in toxicological hazards in the edible products produced from these animals [9]. Furazolidone (FZD), furaltadone (FTD), nitrofurazone (NFZ), and nitrofurantoin (NFT) are commonly used nitrofuran- (NF-) based veterinary drugs [10]. They are typically used to treat gastrointestinal infections in cattle, pigs, and poultry [11]. In addition, many antimicrobial drugs containing NFs are used in the farming of shrimp and fish [12]. However, NFs are prohibited (zero-tolerance policy) in many countries, as initiated by the European Union (EU) in 1993, because of the carcinogenic, teratogenic, and mutagenic potential of NFs, as reported by the World Health Organization (WHO) in 1989 and 1993 [12–15]. The hazardous potential of NFs has been reported for many years [10]. For example, the mutagenicity of NFT and FZD in Chinese hamster ovary cell strains has been examined [16]. It was found that FZD can induce oxidative DNA damage by increasing the number of intracellular reactive oxygen species, thus resulting in the abnormal progression or arrest of the cell cycle in human hepatoma G2 cells [17]. NFZ and FTD have been evaluated toxicologically and show severe toxicity to *Raphidocelis subcapitata*, *Daphnia magna*, and *Musca domestica* [18]. NFs are direct-acting mutagens in the sense that they induce mutations in bacteria and cultures of mammalian fibroblasts without the addition of exogenous activating systems [19]. Because of these findings, the EU has set the minimum required performance level (MRPL) of 1 *μ*g kg^−1^ for each NF [20]. Thus, analytical laboratories must be able to achieve this detection limit, and sensitive and selective methods are required to detect and quantify the residues of these compounds in animal tissues and related food products.

NFMs have been found in products containing SST, but conventional methods are not capable of sufficient analytical accuracy. Thus, we aimed to develop a reliable method to enable the detection and quantification of NFMs in SST food products to enable the better protection of the thousands of people, including children, pregnant women, and the elderly who use these HHFs regularly. Immunoassay techniques have been used for the detection of NFs; however, those methods are problematic due to the interference by other substances in the matrix and the high affinity of immune antibodies having high-risk false-positive and false-negative responses as a fundamental problem methodologically [21, 22]. Although HPLC/UV and HPLC-DAD have long been used for the screening NFs metabolites due to the acceptable reliability, it was lacking specificity as a confirming method, so it could be a costly and time-consuming way [21]. MS analysis offers more improved specificity than immunoassay, HPLC/UV, and HPLC-DAD. Presently, they could be highly sensitive with the best reliability and repeatability. Further, GC-MS is not suitable for routine use in laboratories for chemical detection of NF metabolites [21]. LC-MS/MS analysis is the best choice to screen, determine, and confirm the NF metabolites than those methods in terms of accuracy, precision, sensitivity, and sample preparation. In this article, a simplified and more reliable method was used for the determination of the NF metabolites using LC-MS/MS in soft-shell turtle powder.

The physical and chemical properties of SST powder (SSTP) are as follows: the product has a moisture content of <5%, an ash content of 20–40%, and a crude protein content of 50–70%. These characteristics make SSTP very different from most processed foods [23]. Furthermore, these properties make the analysis of NFMs in the SSTP matrix challenging. For example, conventional testing of SSTP resulted [11, 24–26] in no peaks being detected, even when internal standards (ISs) were used for the mass spectrometry (MS) analysis. Thus, standard analytical experimental conditions are not appropriate for this specific sample, and the method based on the characteristics of SSTP must be developed.

The four most important NFs (FZD, FTD, NFZ, and NFT) are rapidly metabolized into 3-amino-2-oxazolidinone (AOZ), 3-amino-5-morpholinomethyl-2-oxazolidinone (AMOZ), semicarbazide (SEM), and 1-aminohydantoin (AHD), respectively, after administration, and remain as stable protein-bound compounds in animal tissues for long periods [11, 24–26]. Therefore, the majority of methods for analyzing NFs are based on the determination of these highly stable metabolites as markers [11, 27]. However, in some samples, for example, products containing azodicarbonamide treated with chlorine, red seaweed, carrageenan, milk powders, whey powders, egg powder, and wild forest honey from New Zealand, trace amounts of SEM have been detected and are considered environmental contaminants, reaction products formed during processing, or natural substances (for example, in certain seafood) [28, 29]. However, in most samples, detected SEM can only originate from the NFZ used to treat food-producing animals [29]. Thus, we did not exclude SEM from method development. In fact, several analytical methods have been described for the simultaneous detection of NFMs in food products from animals [11, 24–26, 30–33].

In this study, we developed a simultaneous determination method for NFMs in SSTP. The method proposed here is based on liquid chromatography-tandem MS (LC-MS/MS), which has been optimized to achieve the quick, accurate, and sensitive analysis of the complex SSTP matrix. Our method will enable better compliance with HFF food safety policies, which is crucial, considering that the market for these products includes vulnerable groups.

## 2. Materials and Methods

### 2.1. Chemicals and Reagents

Reference standards for AOZ, AMOZ, SEM, and AHD were purchased from Sigma-Aldrich (Steinheim, Germany). Isotopically labeled AOZ-*d*_4_, AMOZ-*d*_5_, ^13^C^15^N_2_·SEM, and AHD-^13^C_3_, which were used as ISs, were also bought from Sigma-Aldrich (Steinheim, Germany). All reference standard purities were over 99.0%. The derivatization reagents 2-nitrobenzaldehyde (2-NBA) and 0.1 M potassium phosphate dibasic solution (K_2_HPO_4_) were purchased from Sigma-Aldrich (Steinheim, Germany). Sodium hydroxide (NaOH, reagent grade) was purchased from Fisher Scientific (Loughborough, Leicestershire, UK). Analytical reagent grade aqueous (37%) hydrogen chloride (HCl) was purchased from Sigma-Aldrich (Steinheim, Germany).

High-performance LC (HPLC) grade ethyl acetate, methanol, and acetonitrile were purchased from Burdick & Jackson (Muskegon, MI, USA). MS analysis grade ammonium formate (AF) and formic acid (FA) solutions were obtained from Sigma-Aldrich (Steinheim, Germany). HPLC grade *n*-hexane and dimethyl sulfoxide (DMSO, analytical grade) were purchased from Merck (Darmstadt, Germany). Deionized ultrapure water was generated using a Milli-Q Plus water purification system (Millipore, Darmstadt, Germany). Water and other mobile-phase solvents were filtered through a 0.2 *μ*m Whatman membrane filter, and a 0.22 *μ*m polytetrafluoroethylene (PTFE) syringe filter was used for samples (Millipore Ltd., Bedford, MA, USA).

### 2.2. Equipment

An Applied Biosystems triple quadrupole mass spectrometer 4500 (AB Sciex, Framingham, MA, USA) was used for analysis with an electrospray ionization (ESI) source operating in positive ion mode. LC was carried out using an Agilent (Santa Clara, CA, USA) 1290 Infinity System. Analyst^®^ (version 1.6.2, AB Sciex Corp., Framingham, MA, USA) was used for method development and data analysis. The data were acquired in multiple reaction monitoring (MRM) mode.

### 2.3. Chromatographic Conditions

The HPLC separation was performed on an Aegispak C18-L (2.0 mm × 100 mm, 5 *μ*m particle size) obtained from Young Jin Biochrom Co., Ltd (Sung Nam, Korea). The temperatures of the column oven and autosampler tray were set to 40 and 4°C, respectively. The mobile phase was composed of solvent A (10 mM AF, 0.1% FA in 20% MeCN/MeOH (1 : 1 v/v)) and solvent B (10 mM AF, 0.1% FA in MeCN/MeOH (1 : 1 v/v)). The injection volume was 20 *μ*L. The flow rate was set at 250 *μ*L mL^−1^, with a linear gradient applied for 10 min to obtain sufficient statistics for each analyte, in the following conditions: 0.0–1.0 min, 100% A; 1.0–2.0 min, 100–0% A; 2.0–7.0 min, 0.0–0.0% A; and 7.1–10.0 min, 0–100% A.

### 2.4. LC-MS/MS Analysis

Stable and common product ions were selected for target analyte identification using syringe injections of the diluted standard solutions of each target analyte at 100 *μ*g L^−1^, respectively. The 2-nitrophenyl (2-NP) derivatized NFMs (2-NP-NFMs) and their ISs were detected in positive-ion mode ESI. The corresponding cone voltage and collision energy under optimal operating conditions, along with the MS/MS transitions used for the analysis, are summarized in Table 1. MS parameter tests were performed for all analytes at an ion source temperature (TEMP) of 600°C and an ionization spray voltage (ISV) of 5500 keV. Nitrogen was used as a nebulizer for the curtain gas (CUR) and collision gas (CG) in accordance with the manufacturer's guidelines. The ion source gas 1 (GS 1, nebulizer gas) and ion source gas 2 (GS 2, turbo gas) flows were set to 30 L h^−1^, and the CUR flow was set to 25 L h^−1^.

### 2.5. Standard Solutions

Standard solutions of NFMs and their IS solutions were prepared by diluting individual stock solutions containing 100 mg L^−1^ of each standard dissolved in MeCN. These solutions were cooled to −20°C in a freezer immediately after preparation. The working standard solution was mixed and then diluted with MeCN to each concentration, i.e., 1.0, 2.5, 5.0, 10.0, 20.0, 50.0, and 100.0 *μ*g L^−1^. The working IS solutions of 200 *μ*g L^−1^ were prepared by mixing and diluting from the stock solution in MeCN. The 2-NBA solution was freshly prepared for use when required at a concentration of 0.05 M in DMSO.

### 2.6. Sample Preparation

Samples were kindly provided by associates in the import trade. The SSTP samples were obtained in two different batches. One batch of SSTP was an uncontaminated clean sample. Another batch of SSTP was contaminated and found to contain 2-NP-AOZ by the screening tests before the study using the conventional analytical method. The sample was an ordinary hot-air-dried powder, and it was stored at room temperature as is typical for this type of foodstuff.

### 2.7. Optimizing the Preparation of SSTP Samples

#### 2.7.1. Sample Weight

**Figure 1:**
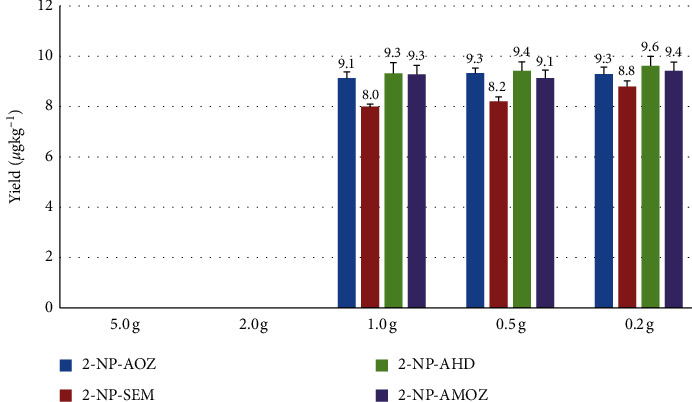
Optimization of sample weight of SSTP obtained by spiking a standard solution with each compound at a concentration of 10 *μ*g kg^−1^. Data are presented as mean ± SD (*n* = 3; *P* < 0.05).

The weight of the SSTP samples can affect the reaction pH value when the hydrolysis and derivatization processes occur. The analyte-free SSTP was used as a blank matrix. To obtain the optimal sample weight, samples weighing 5.0 (conventional sample weight), 2.0, 1.0, 0.5, and 0.2 g were tested, and the results are shown in Figure 1. This analysis was based on the conventional extraction method outlined in the Korean Food Code [34]. This conventional method followed the previously reported hydrolysis and derivatization processes, which require 16 h (overnight) at 37°C [11, 24, 26, 30, 31, 35, 36].

The samples weighed from 5.0 to 0.2 g with a margin of error of 10% of each weight range in 50 mL polypropylene centrifuge tubes. The working standards at 10 *μ*g kg^−1^and working IS solutions were placed in 200 *μ*L volumes into the tubes. The samples were kept for about 15 min at room temperature. Then, a 10 mL aliquot of a 0.125 M HCl solution and a 200 *μ*L aliquot of a 0.05 M 2-NBA solution in DMSO were added to each tube. Then, the tube was vortex-mixed for 0.5 min and kept in a precision reciprocal shaking water bath (Thermo Scientific, NC, USA) for 16 h (overnight) at 37°C in the dark and agitated at 80 rpm. After cooling to room temperature, a 1 mL aliquot of 0.1 M K_2_HPO_4_ was added to each centrifuge tube, and the pH was adjusted to 7.0 ± 0.1. All tubes were centrifuged at 2000 rpm for 10 min in an Avanti J-E centrifuge (Beckman Coulter, Inc., Palo Alto, CA, USA). The upper aqueous layer was transferred to a new tube. Then, 10 mL of hexane was added and vortex-mixed for 30 s. The hexane layer was discarded after centrifugation. Then, 8 mL of EtOAc was added and vigorously vortex-mixed for 30 s. After each sample had been centrifuged at 2500 rpm for 10 min, the supernatant was transferred to a new centrifuge tube. This EtOAc extraction was performed twice.

The extract volume was reduced under a gentle stream of nitrogen at a temperature of less than 40°C using an OA-SYS N-EVAPTM 112 N_2_ evaporator (Organization, Berlin, MA, USA). The dried eluate was diluted with 0.5 mL of the mobile phase and transferred to a 2 mL centrifuge tube and centrifuged at 7500 rpm for 10 min. The clear supernatant was filtered through a 0.22 *μ*m PTFE syringe filter (Millipore Ltd., Bedford, MA, USA) for subsequent HPLC-MS/MS analysis.

### 2.8. Optimization of Reaction Conditions through Direct Use of Incurred SSTP Samples

**Figure 2:**
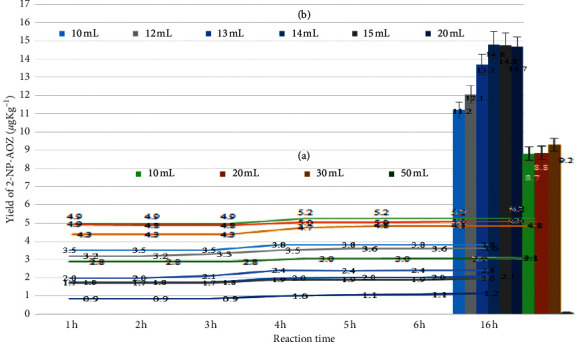
Change in pH over time and amount of 2-NP-AOZ detected in the incurred sample. (a) Concentration of detected 2-NP-AOZ in samples treated with 10, 20, 30, and 50 mL of 0.125 M HCl at 37°C. The results of the experiments with 50 mL were discarded because of errors. (b) Concentration of detected 2-NP-AOZ using 10, 12, 13, 14, 15, and 20 mL of 0.5 M HCl at 45°C. *∗*Line graph: pH value over time (unit: pH). *∗∗* Bar graph: final detected concentration of 2-NP-AOZ in the incurred sample under each test condition (unit: *μ*g kg^−1^).

Using the incurred SSTP samples, the most optimal preparation procedure was obtained. The sample (1.0 g ± 0.1 g) was weighed in 50 mL polypropylene centrifuge tubes with 200 *μ*L of IS solution (200 *μ*g L^−1^), on the basis of the optimal sample weight shown in Figure 1. The change in pH with time was determined by measurements from 1 to 6 h in 1 h intervals and after 16 h using the conditions of the conventional method; the results are shown in Figure 2. The concentration of 2-NP-AOZ in the incurred samples was detected using 10, 20, 30, and 50 mL of 0.125 M HCl at 37°C. The variation in the volume of HCl solutions in the concentration of 0.125 and 0.5 M is shown in Figure 2 and was used to find the optimum volume for each acid concentration. The amount of detected 2-NP-AOZ in the samples is shown in the bar graph in Figure 2.

### 2.9. Reaction Time

**Figure 3:**
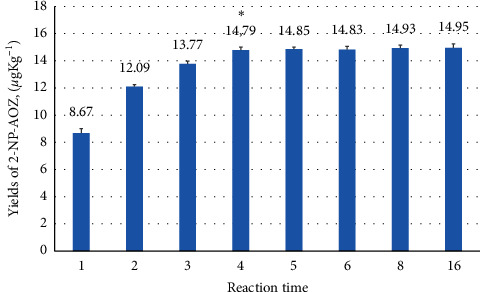
Detected 2-NP-AOZ from the incurred sample over the time under the chosen experimental condition with 14 mL of 0.5 M HCl at 45°C (mean ± SD).

The detection of 2-NP-AOZ was carried out on samples with 10, 12, 13, 14, 15, and 20 mL of 0.5 M HCl at 45°C. Using the results shown in Figure 2, the best time period was determined based on the amount of detected 2-NP-AOZ in the incurred samples. The optimal time period was obtained using a 1.0 g sample with 14 mL of 0.5 M HCl at 45°C, as shown in Figure 3. Other experimental conditions are the same as those reported in the “Sample weight” section of the materials and methods.

### 2.10. Method Validation

The developed method was validated for selectivity, specificity, linearity, limit of detection (LOD), limit of quantitation (LOQ), detection limit (CC*β*), decision limit (CC*α*), and recovery in accordance with the European Commission (EC) regulations [37]. This method was validated on a multiresidue scale for the simultaneous analysis of AOZ, AMOZ, SEM, and AHD as their derivatized nitrophenyl counterparts.

Selectivity and specificity were assessed by analyzing the samples and by testing spiked NFMs in blank samples. The absence of possible interfering compounds around the retention time of the target compounds was verified and compared to those of the spiked samples with the working standard solutions.

Linearity was measured using matrix-matched calibration curves with NF-free SSTP samples at seven different standard concentrations, as shown in Table 2. The calibration curve was obtained from the area ratio of each target compound to the IS concentration to compensate and correct for matrix effects.

The repeatability and reproducibility were tested in three different days by spiking the working standard solution on the blank SSTP.

LOD is usually reported using the signal-to-noise (S/N) ratio [38], in which the signal must be three times higher than the baseline noise. In this study, we define the LOD from the spiked real samples at the lowest standard solution concentration at which a measurement has been carried out [39]. The LOQ was obtained as 10 times the S/N ratios from the spiked standard solutions of blank samples at the lowest concentration. CC*α* and CC*β* were acquired from the testing blank samples and calculated by plotting all data obtained as described in the EC 2002/657/EC [37, 39].

## 3. Results and Discussion

### 3.1. Optimization of SSTP Sample Preparation

#### 3.1.1. Sample Weight

First, the optimal sample weight for the method was determined because of the peculiar characteristics of SSTP samples. When SSTP samples were analyzed using the conventional method, no chromatographic peaks (including IS peaks) were detected, indicating that derivatization did not occur in the SSTP samples with the conventional method. SSTP is a hot-air-dried product that is nutrient and mineral-dense. SSTP is prepared from the whole body, including bones, of SST. Therefore, the lack of reaction is assumed to be a result of the unique characteristics of SSTP. Thus, finding the optimal sample weight is a reasonable first step. The studied sample weights were 0.2, 0.5, 1.0, 2.0, and 5.0 g (conventional sample weight) of SSTP using the fortified standard solution at a concentration of 10 *μ*g kg^−1^.

Presumably, hydrolysis and derivatization reactions do not occur for samples weighing 2.0 to 5.0 g. On the basis of the pH results, a pH around 5.2 or greater cannot induce sufficient hydrolysis and derivatization in the SSTP matrix for detection. At a pH of 5, the density of the mineral content could be the reason for the nonreactiveness, but further research is required to determine this. The peaks could only be observed with efficient recoveries below these weights when using the conventional method. The samples weighing 0.2, 0.5, and 1.0 g yielded similar recoveries for the four target analytes. However, the use of very small samples in the field could introduce errors. Thus, we aimed to balance the use of a truly representative sample without reducing the sample weight excessively. Therefore, a sample weight of 1.0 g was chosen, as shown in Figure 1.

### 3.2. Optimization of Reaction Conditions (Temperature and pH) Using Real Incurred SSTP Samples

Efficient analytical methods for NFMs based on Raman spectrometry and enzyme-linked immunosorbent assays have been investigated but have focused on optimizing the reaction temperature following the published HPLC and LC-MS/MS methods [31–34]. In this part of the study, we aimed to develop a practical, convenient, and facile method for SSTP analysis.

The results of the search for the optimal acid volume and concentration and temperature of the incurred SSTP are shown in Figures 2 and 2. The change in pH during the reaction with 2-NBA was measured over time to optimize the reaction time. The temperature is also a key reaction parameter. The derivatization of NFMs with 2-NBA has been reported to be optimal at 37°C [2, 3, 15–18, 21–24, 26, 35]. However, a recent report shows significant differences (*P* < 0.05) in peak areas of four derivatized NFMs when the reaction temperature exceeded temperatures of 50, 60, and 40°C [36]. Considering earlier works and the results of our pilot study prior to this work, temperatures of 37 and 45°C were used in this study.

The detected concentrations of 2-NP-AOZ in the incurred samples using 10, 20, 30, and 50 mL of 0.125 M HCl at 37°C are shown as a bar graph in Figure 2. The experiment using 50 mL of 0.125 M HCl was stopped during preparation because of excess errors arising from the large volume of the solution. For the remaining samples, the pH value increased slightly with time, but the most noticeable change was from 3 to 5 h, as shown by the line plot. When using 10, 20, and 30 mL of 0.125 M HCl, the pH changed from 4.3 to 5.2. Within this pH range, derivatization and hydrolysis are possible, but the reaction rate could be reduced. In muscle tissue samples, protein hydrolysis and NFM detachment from protein occur simultaneously with an increase in the pH value [32, 40–42].

Most published analytical methods target the analysis of tissues such as unprocessed meat and organs [32, 40–42]. These studies have reported the release of NFMs from the bound state under mildly acidic conditions followed by detection by reaction with 2-NBA to produce the derivatized form [24, 33]. Well-ground muscle tissue is slightly viscous, and proteins could be aggregated when the tissue samples are exposed to excessively strong acid solution without continuous vigorous agitation during the hydrolysis process. This can delay the hydrolysis reaction and prevent the protein from unfolding and releasing NFMs from the tissue for detection. To develop a rapid analysis method, these effects must be carefully considered. SSTP samples are well-dried powders with no viscosity or emulsification during sample preparation. Thus, the concentration of the acid can be increased slightly compared to that used for wet tissue samples.

Consequently, to develop our facile method, 0.5 M HCl and small volumes of the acid solution were tested (Figure 2). In these experiments, the pH increased slightly with time, mainly around 3 to 5 h between pH 0.9 and 3.8. The detected concentrations are not significantly different from those shown in Figure 2. However, the pH was slightly higher for the samples treated with 14, 15, and 20 mL compared to those treated with other volumes. Nevertheless, the pH was lower than those of the samples treated with 0.125 M HCl. The pH of the samples treated with 14, 15, and 20 mL volumes are similar, and the reaction occurred below pH 2.5. Thus, the use of 14 mL of 0.5 M HCl allows easy handling while achieving good performance.

### 3.3. Reaction Time

On the basis of the acquired results, the incurred samples were tested to optimize the reaction period using 14 mL of 0.5 M HCl at 45°C as shown in Figure 3. The amount of 2-NP-AOZ was determined in the samples after 1 to 16 h. At 1, 2, and 3 h, the detected amount increases with an increase in time. However, there was no significant change in the detected amount after 4 h. ANOVA was used for data analysis using OriginPro software (version 8, OriginLab, USA). Significance was set at *P* < 0.05, and the results are expressed as mean ± SD. Previously published methods have mainly used 16 h (overnight) for hydrolysis and derivatization with 2-NBA [2, 21, 23, 27, 28, 32, 33]. However, in our experiments, 4 h was sufficient reaction time and was selected as the optimum reaction period.

### 3.4. Sample Treatment Optimization

SSTP samples contain bound fatty acids, minerals, and other nutrients. To remove lipids from the SSTP samples, a single defatting process is not sufficient. However, the addition of hexane washing steps or the use of solid-phase extraction (SPE) will reduce the ease, simplicity, and cost-effectiveness of the method. Consequently, the eluate was reconstituted with 0.5 mL of mobile phase A and transferred to a 2 mL centrifuge tube and centrifuged at 11,500 rpm for 20 min at 0°C to remove lipids by freezing. Then, the clear supernatant was filtered through a 0.22 *µ*m PTFE syringe filter.

Concerning the abundant mineral content of the samples, centrifugation was used to isolate the aqueous layer after the derivatization and hydrolysis processes. The addition of EtOAc allowed the extraction of the target NFMs from the aqueous layer and accelerated mineral precipitation, which is visible by the naked eye after centrifugation.

### 3.5. Optimization of LC-MS/MS Conditions

Optimization of the MS parameters was carried out by performing a series of syringe injections with diluted standard solutions of the derivatized NFMs (100 *μ*g L^−1^) with 50% mobile phase B using ESI + as the ionization source. The analysis was carried out with Analyst (version 1.6.2) to determine the parent ions and the optimal fragment voltage for syringe injections of the tuning solution. The optimized fragment voltage was then chosen, and the precursor ion was fragmented and collected according to the change in collision energy, where the product ions were selected by the software. The most abundant ion was selected for quantification purposes. Two other transition ions were chosen for identification. MRM was used to evaluate the performance of this method with positive electrospray ionization. Each analyte was examined in ESI + mode by monitoring the selected reaction. The most abundant and stable product ions were chosen to identify the target analytes. Table 1 lists the MRM transitions, individual declustering potentials, and collision energy voltages used in the analysis. Then, MS parameter tests were performed for all analytes at an ion source temperature (TEMP) of 600°C, an ionization spray voltage (ISV) of 5500 keV, a curtain gas (CUR) pressure of 25 kPa, a collision gas pressure of 8 kPa, an ion source gas 1 (GS 1, nebulizer gas) a pressure of 40 kPa, and an ion source gas 2 (GS 2, turbo gas) pressure of 45 kPa. The MS/MS transitions and transition ratios for derivatives of the NFMs are presented in Table 1.

### 3.6. Method Validation

Method validation was conducted based on EC regulations 2002/657/EC [37], as summarized in Table 2. The selectivity, specificity, and sensitivity were evaluated and were consistent with EC regulations 2002/657/EC, as shown by the good results (including at a concentration of 1 *μ*g kg^−1^), as shown in Table 2. The linearity was evaluated using calibration curves constructed using a blank sample spiked with the target NFMs. All analytes exhibit excellent linearity in the studied concentration range, having correlation coefficients higher than 0.999. The S/N ratio was determined by comparison with the signals with the target analytes of the lowest concentration and the blank samples. The LOD and LOQ were determined from the minimum concentration, i.e., 3 and 10 times larger than the S/N, respectively [39]. The CC*α* and CC*β* values were also acceptable: 0.18–0.30 and 0.30–0.34, respectively. The accuracy and precision were verified for each NFM based on their intraday and interday variabilities. The accuracy and precision were identified with each recovery and percentage root square deviation (% RSD) and determined in terms of the peak areas at four concentration levels (0.5, 1.0, 2.0, and 10.0 *μ*g kg^−1^) assuming an acceptability criterion of ±15%.

**Figure 4:**
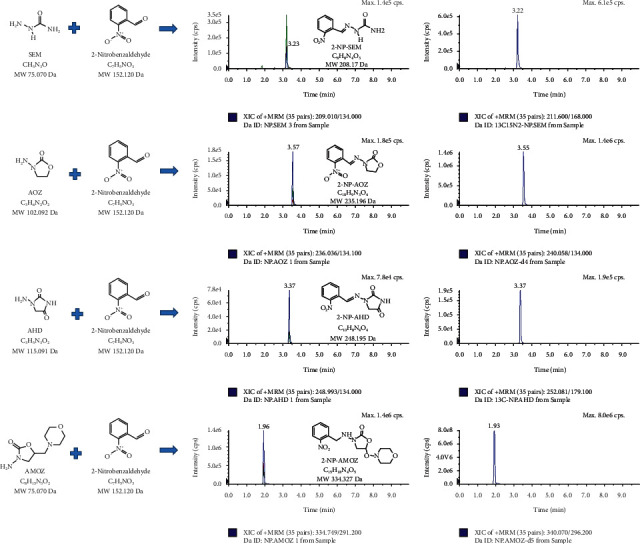
Chromatograms of four NF metabolites (AOZ, AMOZ, AHD, and SEM (10 *μ*g kg^−1^)) and the ISs in the SSTP samples.

The intraday and interday accuracy were 82.2–102.5% and 85.2–108.1%, respectively, for all analytes. The ranges of interday mean recovery for each analyte were 92.8–101.3% for 2-NP-AHD, 92.6–98.2% for 2-NP-SEM, 85.2–108.1% for 2-NP-AOZ, and 90.4–96.5% for 2-NP-AMOZ. The result values are consistent with the legislated MRPLs for the four NFMs [40]. Chromatograms of the SSTP matrices spiked with NFMs and ISs are shown in Figure 4. Thus, our developed method enables rapid analysis of the SSTP matrix with reliable sensitivity, which has not been previously reported.

### 3.7. Findings in Samples Obtained from Local Markets

To verify the utility of the method developed in this work, the method was applied for the determination of illegal NFs in real HFF samples. SSTP is typically encapsulated and used as an ingredient in HFFs. SST is believed to increase longevity in Asia and known to be highly nutritious, so, as a healthy food, it is traded at a high price. Primarily, it is sold to promote the health of the elderly and the growth of young children. Thirteen HFF products obtained from Internet sellers were analyzed. SEM and AOZ were detected in three samples, as shown in Table 3. In one sample (R4), SEM was detected below the LOQ. SEM was detected in three samples (C11, C12, and C13) at concentrations of 12.13, 1.02, and 0.54 *μ*g kg^−1^, respectively, with reasonable precision. AOZ was detected in one sample (C11) at a concentration of 9.30 *μ*g kg^−1^ with excellent precision.

The results of the analysis of real product samples show that our method is suitable for the simultaneous determination of illegal NFs in HFF by routine analysis. However, as is typical of HFFs, these residues are consumed in capsule form regularly (e.g., three times a day), and there is no information regarding effects on consumers exposed to this regular pattern, which will be different from the irregular consumption of aquatic produces or meat containing residues after cooling. Thus, further research is needed in this area.

Finally, as discussed in the Introduction, proper sample preparation is key to accurate determination because of the wide sample variety. As shown in Table 3, our method can be successfully applied to commercially available HHF samples.

## 4. Conclusion

By using incurred SSTP samples, a fast and robust LC-MS/MS method has been developed for the simultaneous determination of NFMs in the unique, challenging SSTP matrix. The method reduced the reaction time required for derivatization and hydrolysis from 16 to 4 h using simple demineralization and freezing-defatting steps and achieved desirable reliability, sensitivity, simplicity, and time efficiency in the total test process.

The present method was applied to real SSTP samples, and the result demonstrates the strong detection and quantification of NF marker residues. Furthermore, the method is fully compliant with the recommended guidelines established by the EC regulation 2003/181/EC, as shown by experiments using real contaminated SSTP samples. In summary, this developed method is a simple, efficient, and powerful tool to improve food safety in a current blind spot in food industry regulation.

## Figures and Tables

**Table 1:** Mass spectrometry parameters for the optimal operational analysis of the derivatized NF metabolites.

Analytes	Retention time (min)	Precursor ion (*m/z*)	Product 1 ion (*m/z*)	DP/CE/CXP (eV)	Product 2 ion (*m/z*)	DP/CE/CXP (eV)	Product 3 ion (*m/z*)	DP/CE/CXP (eV)
2-NP-AHD	3.37	248.9	134.0*∗*	106/17/10	178.1	106/21/8	104.0	106/27/8
2-NP-AOZ	3.57	236.0	134.1*∗*	116/17/8	149.0	116/19/12	104.0	116/33/12
2-NP-AMOZ	1.95	334.7	291.2*∗*	96/17/8	262.1	96/23/10	128.1	96/27/10
2-NP-SEM	3.23	209.0	166.1*∗*	10/13/8	134.0	66/15/8	192.0	66/17/8
2-NP-^13^C^15^N_2_·SEM	3.22	211.6	168.0	56/15/8	ㅡ	ㅡ	ㅡ	ㅡ
2-NP-^13^C_3_-AHD	3.37	252.0	179.1	61/21/6	ㅡ	ㅡ	ㅡ	ㅡ
2-NP-AOZ-d4	3.55	240.0	134.0	111/17/10	ㅡ	ㅡ	ㅡ	ㅡ
2-NP-AMOZ-d5	1.93	340.0	296.2	46/17/14	ㅡ	ㅡ	ㅡ	ㅡ

RT: retention time (min); DP: declustering potential (electron volt); CE: collision energy (electron volt); CXP: collision cell exit potential (electron volt). *∗* Transitions for quantifier peaks.

**Table 2:** Calibration curves, sensitivity, corrected intraday and interday accuracies, and precision for samples spiked with working standard solution (*n* = 6).

Target analyte	Linearity range (*μ*g kg^−1^)	Spiked level (*μ*g kg^−1^)	Calibration curve equation (*r*)	Intraday series (% RSD)	Interday series (% RSD)	LOD (*μ*g kg^−1^)	LOQ (*μ*g kg^−1^)	CC*α* (*μ*g kg^−1^)	CC*β* (*μ*g kg^−1^)
2-NP-AHD	0.2–20	0.5	*y* = 0.0124*x* + 0.1991 (0.99969)	98.8 (3.2)	92.8 (3.6)	0.07	0.23	0.25	0.30
		1.0		96.9 (3.2)	101.3 (3.8)				
		2.0		102.5 (2.1)	98.3 (2.9)				
		10.0		98.7 (1.7)	99.4 (2.5)				

2-NP-SEM	0.2–20	0.5	*y* = 0.0477*x* + 0.1280 (0.99971)	95.5 (3.8)	92.6 (4.5)	0.05	0.17	0.18	0.30
		1.0		92.5 (3.1)	98.2 (3.5)				
		2.0		94.8 (2.1)	92.7 (3.8)				
		10.0		95.2 (2.2)	93.9 (2.8)				

2-NP-AOZ	0.3–20	0.5	*y* = 0.0127*x* + 0.2459 (0.99941)	82.2 (3.7)	85.2 (4.8)	0.09	0.29	0.30	0.34
		1.0		84.7 (3.2)	88.5 (3.4)				
		2.0		87.5 (2.4)	108.1 (2.8)				
		10.0		90.4 (2.8)	87.6 (3.1)				

2-NP-AMOZ	0.2–20	0.5	*y* = 0.0235*x* + 0.2192 (0.99980)	92.6 (2.2)	90.4 (3.1)	0.05	0.17	0.27	0.32
		1.0		85.6 (3.4)	93.3 (3.7)				
		2.0		93.0 (2.3)	94.8 (3.4)				
		10.0		95.6 (1.5)	96.5 (2.2)				

**Table 3:** Detection of NF metabolites in HHF products purchased online.

	AOZ			AHD			SEM			AMOZ		
	CON (*μ*g kg^−1^)	STV	% RSD	CON (*μ*g kg^−1^)	STV	% RSD	CON (*μ*g kg^−1^)	STV	% RSD	CON (*μ*g kg^−1^)	STV	% RSD
R1	ND	ND	ND	ND	ND	ND	ND	ND	ND	ND	ND	ND
R2	ND	ND	ND	ND	ND	ND	ND	ND	ND	ND	ND	ND
R3	ND	ND	ND	ND	ND	ND	ND	ND	ND	ND	ND	ND
R4	ND	ND	ND	ND	ND	ND	BL	BL	BL	ND	ND	ND
R5	ND	ND	ND	ND	ND	ND	ND	ND	ND	ND	ND	ND
R6	ND	ND	ND	ND	ND	ND	ND	ND	ND	ND	ND	ND
R7	ND	ND	ND	ND	ND	ND	ND	ND	ND	ND	ND	ND
R8	ND	ND	ND	ND	ND	ND	ND	ND	ND	ND	ND	ND
R9	ND	ND	ND	ND	ND	ND	ND	ND	ND	ND	ND	ND
R10	ND	ND	ND	ND	ND	ND	ND	ND	ND	ND	ND	ND
C11	9.30	0.17	1.86	ND	ND	ND	12.13	0.23	1.90	ND	ND	ND
C12	ND	ND	ND	ND	ND	ND	1.02	0.04	3.70	ND	ND	ND
C13	ND	ND	ND	ND	ND	ND	0.54	0.02	2.85	ND	ND	ND

ND: not detected; BL: below the LOQ; CON: concentration; STV: standard deviation.

## Data Availability

The data used to support the findings of this study are included within the article.
